# scTML: a pan-cancer single-cell landscape of multiple mutation types

**DOI:** 10.1093/nar/gkae898

**Published:** 2024-10-18

**Authors:** Haochen Li, Tianxing Ma, Zetong Zhao, Yixin Chen, Xi Xi, Xiaofei Zhao, Xiaoxiang Zhou, Yibo Gao, Lei Wei, Xuegong Zhang

**Affiliations:** MOE Key Lab of Bioinformatics, Bioinformatics Division of BNRIST and Department of Automation, Tsinghua University, 30 Shuangqing Rd, Haidian District, Beijing 100084, China; School of Medicine, Tsinghua Medicine, Tsinghua University, 30 Shuangqing Rd, Haidian District, Beijing 100084, China; MOE Key Lab of Bioinformatics, Bioinformatics Division of BNRIST and Department of Automation, Tsinghua University, 30 Shuangqing Rd, Haidian District, Beijing 100084, China; MOE Key Lab of Bioinformatics, Bioinformatics Division of BNRIST and Department of Automation, Tsinghua University, 30 Shuangqing Rd, Haidian District, Beijing 100084, China; Department of Biostatistics, School of Public Health, Yale University, 60 College St, New Haven, CT 06510, USA; MOE Key Lab of Bioinformatics, Bioinformatics Division of BNRIST and Department of Automation, Tsinghua University, 30 Shuangqing Rd, Haidian District, Beijing 100084, China; MOE Key Lab of Bioinformatics, Bioinformatics Division of BNRIST and Department of Automation, Tsinghua University, 30 Shuangqing Rd, Haidian District, Beijing 100084, China; MOE Key Lab of Bioinformatics, Bioinformatics Division of BNRIST and Department of Automation, Tsinghua University, 30 Shuangqing Rd, Haidian District, Beijing 100084, China; Department of Thoracic Surgery, National Cancer Center/National Clinical Research Center for Cancer/Cancer Hospital, Chinese Academy of Medical Sciences and Peking Union Medical College, No. 17 Panjiayuan Nanli, Chaoyang District, Beijing 100021, China; Department of Thoracic Surgery, National Cancer Center/National Clinical Research Center for Cancer/Cancer Hospital, Chinese Academy of Medical Sciences and Peking Union Medical College, No. 17 Panjiayuan Nanli, Chaoyang District, Beijing 100021, China; Institute of Cancer Research, Henan Academy of Innovations in Medical Science, No. 2 Biotechnology Street, Hangkonggang District, Zhengzhou 450000, China; Department of Gastroenterology, Shanxi Province Cancer Hospital/Shanxi Hospital Affiliated to Cancers Hospital, Chinese Academy of Medical Sciences/Cancer Hospital Affiliated to Shanxi Medical University, No. 3，ZhiGongXin Street, Xinghualing District, Taiyuan 030013, China; Central Laboratory and Shenzhen Key Laboratory of Epigenetics and Precision Medicine for Cancers, National Cancer Center/National Clinical Research Center for Cancer/Cancer Hospital and Shenzhen Hospital, Chinese Academy of Medical Sciences and Peking Union Medical College, 113 Baohe Road, Longgang District, Shenzhen 518116, China; Laboratory of Translational Medicine, National Cancer Center/National Clinical Research Center for Cancer/Cancer Hospital, Chinese Academy of Medical Sciences and Peking Union Medical College, No. 17 Panjiayuan Nanli, Chaoyang District, Beijing 100021, China; State Key Laboratory of Molecular Oncology, National Cancer Center/National Clinical Research Center for Cancer/Cancer Hospital, Chinese Academy of Medical Sciences and Peking Union Medical College, No. 17 Panjiayuan Nanli, Chaoyang District, Beijing 100021, China; MOE Key Lab of Bioinformatics, Bioinformatics Division of BNRIST and Department of Automation, Tsinghua University, 30 Shuangqing Rd, Haidian District, Beijing 100084, China; MOE Key Lab of Bioinformatics, Bioinformatics Division of BNRIST and Department of Automation, Tsinghua University, 30 Shuangqing Rd, Haidian District, Beijing 100084, China; School of Medicine, Tsinghua Medicine, Tsinghua University, 30 Shuangqing Rd, Haidian District, Beijing 100084, China

## Abstract

Investigating mutations, including single nucleotide variations (SNVs), gene fusions, alternative splicing and copy number variations (CNVs), is fundamental to cancer study. Recent computational methods and biological research have demonstrated the reliability and biological significance of detecting mutations from single-cell transcriptomic data. However, there is a lack of a single-cell-level database containing comprehensive mutation information in all types of cancer. Establishing a single-cell mutation landscape from the huge emerging single-cell transcriptomic data can provide a critical resource for elucidating the mechanisms of tumorigenesis and evolution. Here, we developed scTML (http://sctml.xglab.tech/), the first database offering a pan-cancer single-cell landscape of multiple mutation types. It includes SNVs, insertions/deletions, gene fusions, alternative splicing and CNVs, along with gene expression, cell states and other phenotype information. The data are from 74 datasets with 2 582 633 cells, including 35 full-length (Smart-seq2) transcriptomic single-cell datasets (all publicly available data with raw sequencing files), 23 datasets from 10X technology and 16 spatial transcriptomic datasets. scTML enables users to interactively explore multiple mutation landscapes across tumors or cell types, analyze single-cell-level mutation-phenotype associations and detect cell subclusters of interest. scTML is an important resource that will significantly advance deciphering intra-tumor and inter-tumor heterogeneity, and how mutations shape cell phenotypes.

## Graphical Abstract

**Graphical Abstract:**
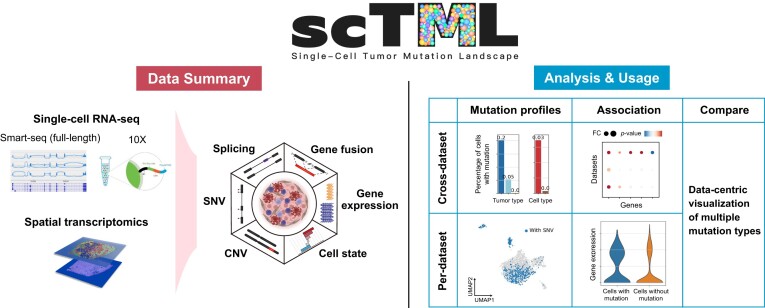

## Introduction

Multiple types of mutations such as single nucleotide variations (SNVs), gene fusions, alternative splicing and copy number variations (CNVs) are common in tumors. These mutations play important roles in tumor occurrence and progression, and compose the intra-tumor and inter-tumor heterogeneity, often leading to different immune microenvironments (1–3). Studying these mutation types and their cellular context is of great significance for investigating the key mechanisms of tumorigenesis and selecting treatment plans accordingly.

The emerging huge amount of single-cell transcriptomic data and atlas projects comprehensively characterize diverse cell types and states in various normal and tumor tissues (4–6). Calling mutations from single-cell transcriptomic data can link genetics to cellular molecular traits in large-scale data without the need for complex and nonuniform experimental protocols for joint profiling of the DNA and RNA from the same cell. Recent computational methods such as scFusion (7), Cellsnp-lite (8), SComatic (9), SCmut (10), Monopogen (11), scReadCounts (12), BRIE (13) and STmut (14), have verified the reliability of detecting multiple types of mutations from single-cell transcriptomic data. Biological research has also demonstrated the biological significance. Du *et al.* detected the subcluster of leukemic blasts with both NUP98/NSD1 and RUNX1/ZNF423 fusion (15). Muñoz *et al.* revealed splicing variations among some single macrophages leading to different functions (16). Single-cell-level SNV profiles from single-cell transcriptomic data demonstrated their significance in distinguishing tumor cell subpopulations, characterizing tumor cell phenotypes, and lineage tracing (17–19). The combined analysis of mutations and gene expression on individual cells has yielded many biological discoveries (7,20–23).

Establishing a single-cell mutation landscape can provide a critical resource for studying the association between mutations and their gene expression profiles, and for elucidating the mechanisms of tumorigenesis and evolution. However, there is a lack of a single-cell-level database that contains comprehensive mutation information. Existing databases such as scQTLbase (24) and CancerSCEM (25) only provide bulk-level mutation information with single-cell gene expression profiles, which limits our understanding of mutations in heterogeneous cell populations and how mutations shape cell phenotypes.

Therefore, we built scTML (http://sctml.xglab.tech/), the first comprehensive pan-cancer single-cell mutational landscape database. From around 80T raw sequencing data, we called multiple types of mutations at the single-cell level, including SNVs, insertions/deletions, gene fusions, alternative splicing and CNVs, along with gene expression, cell states and other phenotype information. The data are from 74 datasets with 2 582 633 cells, including datasets from full-length (Smart-seq2), 10X and spatial transcriptomic technology. We collected all publicly available single-cell full-length transcriptomic data with raw sequencing reads and established the analysis framework in scTML primarily based on full-length transcriptomic data, considering their significantly higher coverage and mutation detection rates. Additionally, we provide the mutation profiles of 10X and spatial transcriptomic data to better utilize their larger data volumes to establish a more comprehensive mutational landscape. Besides the rich information and valuable resources in scTML, we also enable users online exploring the landscapes of multiple mutations across tumors or cell types, analyzing single-cell-level mutation-phenotype relationships and detecting cell subclusters of interest.

## Materials and methods

### Data collection and preprocessing

This article collects tumor full-length single-cell transcriptomic data from Gene Expression Omnibus (GEO) (26), Sequence Read Archive (SRA) (27), European Nucleic Archive (ENA) (28) and Genome Sequence Archive (GSA) (29). The search keywords are combinations of ‘scRNA-seq’, ‘single-cell’, ‘transcriptomics’, ‘Smart-seq2’, ‘full-length’, ‘tumor’ and ‘cancer’. After obtaining the candidate dataset list, we manually read the metadata of each dataset to ensure that it meets the following conditions: (i) it is tumor single-cell transcriptomic data based on full-length sequencing technologies such as Smart-seq2; (ii) original sequencing reads can be obtained; (iii) the number of original cells in the dataset should not be <100. Finally, a total of 35 eligible full-length datasets were obtained (Supplementary Table S1). We collected the public 10X and spatial transcriptomic data according to atlas research and databases (30–32). We required that the raw sequencing reads can be obtained. We finally collected 23 datasets from the 10X technology and 16 spatial transcriptomic datasets (Supplementary Table S1).

We downloaded the raw sequencing reads (fastq or bam files) of these data. We also manually read metadata to label the tumor type, tumor source, and whether the tumor is primary or metastatic. Tumor sources are divided into four categories, including tissue, cell line, patient-derived tumor xenograft (PDX) and circulating tumor cells (CTCs).

### Gene expression quantification and data quality control

Based on obtained the raw sequencing reads, we quantified gene expression. Firstly, based on STAR (33), we align full-length transcriptomic sequencing reads to the hg19 human reference genome to obtain the genomic coordinates of the sequencing reads. Then, based on human gene annotation (GENCODE19 (34)), the featureCounts (35) algorithm was used to count the number of sequencing reads for each gene region, and transcripts per million (TPM) was calculated. Finally, gene expression was quantified as log2 (TPM + 1). For 10X single-cell and spatial transcriptomic datasets, we applied CellRanger and SpaceRanger for quantifying gene expression.

Then, we conduct quality control on each dataset, retaining only cells that meet the following conditions: (i) the number of uniquely mapped reads is not <1 × 10^4^; (ii) the proportion of reads uniquely mapped on mitochondrial genes is <30%; (3) the number of expressed genes is >200. For each dataset, only genes expressed in no <3 cells are retained.

### SNV detection and gene fusion calling

After sequence alignment of single-cell full-length transcriptome sequencing reads, this study uses GATK (36) to detect SNVs from the aligned data. The mutation reference data used (dbSNP138 (37) and 1000G phase 1 (38)) were obtained from the official GATK website. Considering the calling speed and quality, we used cellsnp-lite (8) mode 1a in 10X single-cell and spatial transcriptomic datasets. We annotated SNVs based on ANNOVAR (39) and mainly included SNVs causing amino acid changes in the analysis framework. We used STAR-Fusion (40) to detect gene fusion events from raw sequencing reads.

### Quality control for SNV and gene fusion calling

For SNV, we followed the GATK pipeline and removed mutations with QualByDepth (QD) <2.0 or FisherStrand (FS) >30.0. QD is the variant confidence divided by the unfiltered depth of non-hom-ref samples. According to the GATK documentation, for filtering purposes, QD is better than either variant confidence or depth directly. FS is the Phred-scaled probability that there is strand bias at the site. An FS value close to 0 means nearly no strand bias (whether the alternate allele was seen often on the forward or reverse strand) at the site. Also, we only kept SNVs with records in dbSNP (37) and having a rsID, to make the SNVs included in scTML reliable SNVs verified before.

For gene fusion, we only retained gene fusion events with a fusion fragment count no < 0.1 per million sequencing reads.

### Alternative splicing quantification

We used BRIE (13) to quantify the percentage spliced in (PSI) of variable splicing exons based on aligned sequencing reads. BRIE is a variable splicing quantification algorithm based on Bayesian models combining prior knowledge related to variable splicing with single-cell observations. A larger PSI means a higher proportion of the exon retained in the transcript.

### Cell type annotation

We comprehensively annotated the cells in each dataset using metadata information and marker genes. According to the tumor type, we determined potential cell types (e.g. hepatocytes cannot appear in central nervous system tumors). If some datasets are from tumor cell lines, the cell type can be directly limited to tumor cells.

Then, we performed high variable gene detection, principal component analysis, and clustering based on the Leiden algorithm (setting resolution as 1.0). All calculations are based on the default parameters of Scanpy (41).

We collected and organized marker genes from literature (25,42,43) (Supplementary Table S2). Considering the characteristics of Smart-seq2 datasets, and the consistency among various cancer types and data types, we designed the following cell type annotation strategy according to previous Smart-seq2 research such as Karaayvaz *et al.* and Li *et al.* (44,45), and manually checked the annotation results based on the provided annotation results of some data.

For a certain cell cluster *C*, it is assumed that there are *k* possible cell types. We defined the cell-type possibility score of this cell cluster as *s_1_, s_2_, ⋯, s_k_*. The possibility score *s_i_* of cell type *i* represents the number of cells in this cluster that express cell type *i* marker genes (Supplementary Table S2). The possibility score *s_i_* for cell type *i* is defined as:


\begin{eqnarray*}{{s}_i} = \mathop \sum \limits_{c \in C} {{T}_i}\left( c \right)\end{eqnarray*}

where *c* represents the cells in cell cluster *C*, and *T_i_(c)* represents whether the cell expresses the marker gene for cell type *i*. For cell *c*, if the average expression level of marker genes for cell type *i* is >1, then T_i_(c) = 1. Otherwise, T_i_(*c*) = 0. After obtaining the *k* cell-type possibility scores of this cell cluster, we defined the cell type with the highest score as the cell type of the cluster.

### CNV quantification and malignant cell identification

We identified the malignant-cell candidate cell type according to the tumor type. For example, in colorectal cancer, lung cancer and other solid tumors, the malignant-cell candidate cell type is epithelial cells. For glioma, the candidate cell type is astrocytes or oligodendrocytes. For multiple myeloma, the candidate cell type is plasma cells.

We used InferCNVpy (46) to calculate the CNV value by comparing the sum of all gene expression levels in a genomic region between the target cell and the reference cell. There are 4866 genomic regions and corresponding 4866 CNV values for each cell. We set immunocytes or stroma cells as the reference cells, and calculated the CNV matrix and final CNV score using InferCNVpy for each cell of malignant-cell candidate cell type. We cannot calculate CNV scores in datasets without any immunocytes or stroma cells.

Then, we used InferCNVpy to cluster cells based on the CNV matrix and labeled cell clusters with significantly higher average CNV scores than reference cell types as malignant cells.

### Cell state quantification

Based on MSigDB (47) and Gene Ontology (48), CancerSEA (42) provided 14 cell states of tumor cells, including angiogenesis, apoptosis, cell cycle, differentiation, DNA damage, DNA repair, epithelial–mesenchymal transition (EMT), hypoxia, inflammation, invasion, metastasis, proliferation and quiescence. We collected signature genes for each cell state from CancerSEA and used GSVA (49) to calculate the cell state scores for malignant cells.

### Statistical analyses

The differential analysis for cross-dataset association is based on the ‘rank_genes_groups’ function (Wilcoxon) with default parameters in Scanpy (v1.9.6) (41). Other analyses are based on Pandas (v1.3.5), Numpy (v1.22.4) and Scipy (v1.11.4).

### Database construction

The online database framework deployed on NGINX and Gunicorn (v22.0.0) in an Ubuntu environment. Some results are generated instantly when users query and transmit using Fastapi (v0.111.0) and Uvicorn (v0.29.0). Front-end packages such as jQuery (v3.4.1), echarts (v5.5.0), zingchart (v2.8.9) and select2 (v4.1.0) were utilized for the visualization of results.

## Results

### Overview of scTML

**Figure 1.**
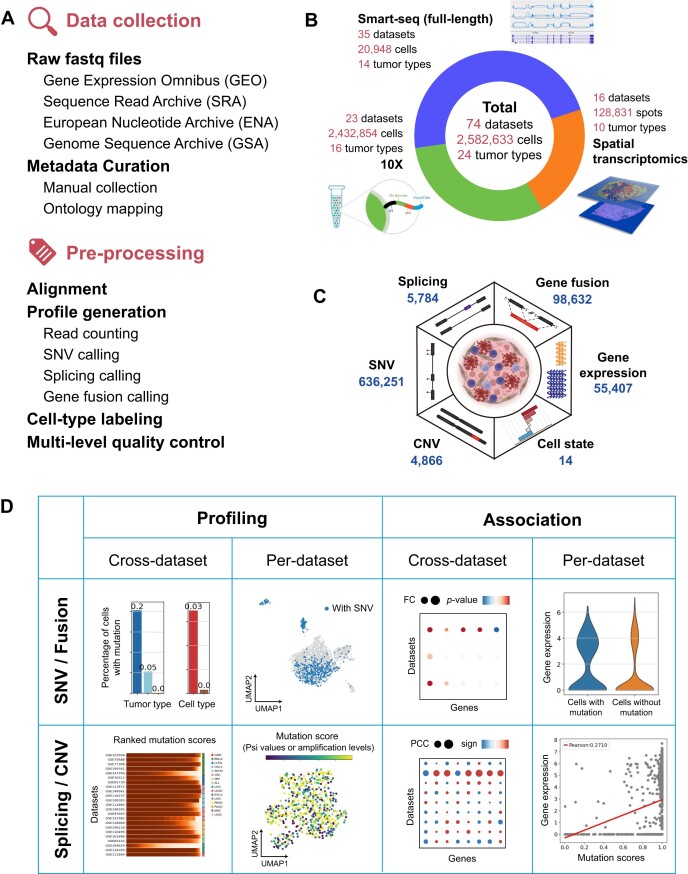
Overview of scTML. (**A**) The procedure of data collection and pre-processing. (**B**) The data composition of scTML. (**C**) The mutation types included in scTML. (**D**) The analysis framework of scTML. The mutation types can be classified into two categories: discrete (SNVs and gene fusions) and continuous (alternative splicing and CNVs). ‘Profiling’ and ‘Association’ correspond to two web pages, respectively.

scTML offers a pan-cancer single-cell landscape of multiple mutation types for investigating single-cell-level mutation patterns and exploring mutation-phenotype associations. We collected 74 single-cell transcriptomic datasets with 2 582 633 cells in total, covering 24 tumor types. We collected all 35 full-length datasets with publicly available raw sequencing files, 23 datasets from 10X technology and 16 spatial datasets. We integrated existing mapping and mutation-calling methods, forming a unified computational framework and calling mutations from around 80T raw sequencing data (Figure 1A and B). scTML includes 636 251 SNVs and insertions/deletions, 98 632 gene fusions, 5784 alternative splicing and 4866 CNVs, along with 55 407 gene expression, 14 cell states and other phenotype information (Figure 1C).

Based on these resources, we designed the analysis framework from four aspects: ‘cross-dataset profiling’, ‘per-dataset profiling’, ‘cross-dataset association’ and ‘per-dataset association’. Our analysis framework is primarily based on full-length transcriptomic data, considering their significantly higher coverage and mutation detection rates. The mutation types can be classified into two categories: discrete (represented by the presence or absence in a cell, including SNVs and gene fusions) and continuous (represented by a score in a cell, including alternative splicing and CNVs) (Figure 1D). For cross-dataset profiling, we calculated the percentage of cells with the selected discrete mutation in each tumor type and cell type, and for continuous mutations, we visualized the distribution of mutation scores by arranging their scores in order and compared the distribution among datasets. For per-dataset profiling, we calculated UMAP projection based on gene expression in each dataset, and then plotted the selected mutation on the UMAP. For cross-dataset association, we calculated the gene differential expression analysis between the cells with and without the selected discrete mutation in each dataset, and calculated the Pearson’s correlation coefficient (PCC) for continuous mutations. Using bubble plots, we represented the association direction in dot color (signed logarithmic adjusted *P*-value or the sign of PCCs) and represented the degree of association in dot size (the absolute value of logarithmic fold change or PCCs). As for the per-dataset association, we plotted the association in the selected dataset using violin plots for discrete mutations and scatter plots with the regression line for continuous mutations (Figure 1D).

We then displayed results from the analysis framework on three web pages: ‘Mutation profile’, ‘Association’ and ‘Compare’. The first page mainly includes cross-dataset and per-dataset profiling. The second page mainly includes cross-dataset and per-dataset associations. In the ‘Compare’ page, we put the profiles of different mutation types from the same dataset together and provided a dataset-centric presentation, considering that the first two pages both selected a mutation first.

### Explore the cross-dataset and per-dataset mutation profiles

On the ‘Mutation profile’ page, users can explore the cross-dataset and per-dataset mutation profiles. This page provides three modules: ‘Mutation profile summary’, ‘Selecting one mutation for viewing the cross-data profiling’ and ‘Viewing mutation in one dataset’.

**Figure 2.**
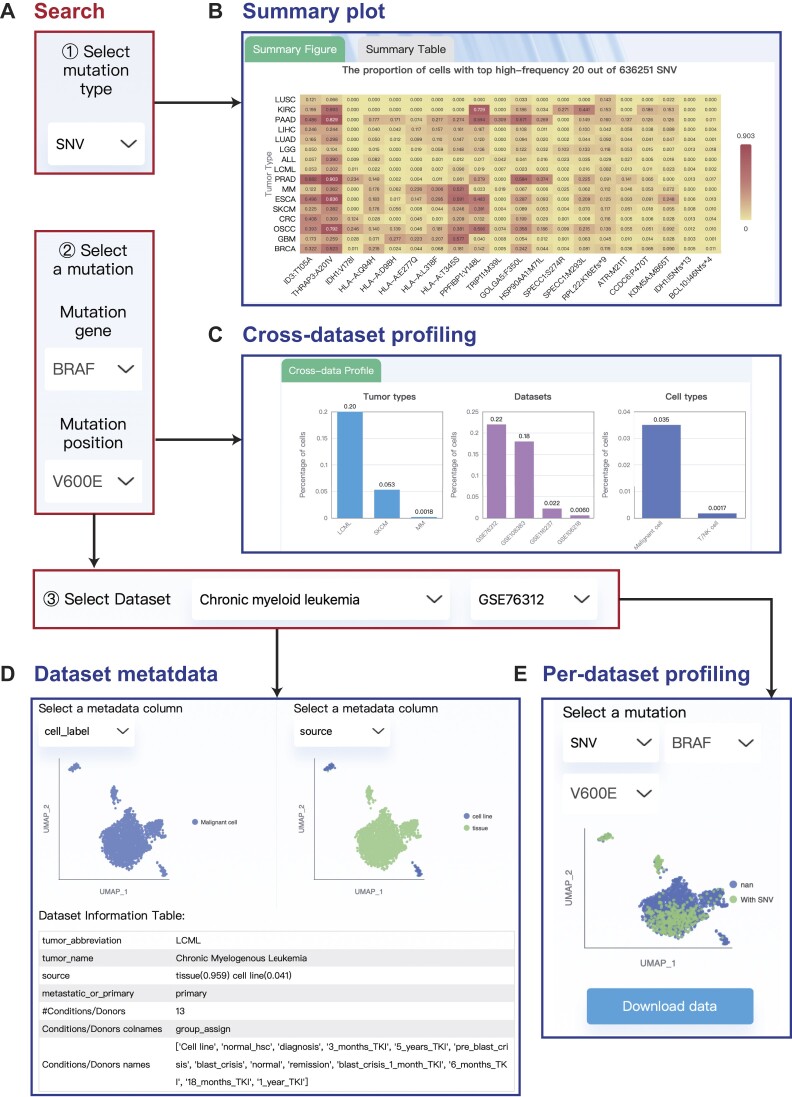
The web interface for mutation profiling in scTML. (**A**) Query interface for selecting a mutation type, a specific mutation, and a dataset. (**B**) An example of mutation summary plots after selecting the mutation type SNV, showing the top 20 SNVs with the highest average cell proportion in each tumor type. (**C**) An example of cross-dataset profiling after selecting the *BRAF* V600E mutation, showing its cell percentage in each dataset, tumor type, and cell type. (**D**) The dataset metadata table and the UMAP plot with metadata labels after selecting the chronic myeloid leukemia GSE76312 dataset. (**E**) An example of per-dataset profiling, showing the UMAP of *BRAF* V600E in the GSE76312 dataset.

As the first step, users need to select a mutation type among four types (Figure 2A). Then, users can view the mutation summary heatmap, which shows the top 20 SNVs (or fusions) with the highest average cell proportion in each tumor type (Figure 2B). For alternative splicing (or CNVs), the heatmap shows the average standard deviation of Psi values for each alternative splicing transcript (or amplification levels for chromosome segments).

As the second step, taking the mutation type SNV as an example, users can select a mutation gene, and then select an amino acid change. Then, the cross-data profiling plot can show the cell percentage of this mutation in each dataset, tumor type and cell type (Figure 2C). When selecting alternative splicing or CNVs, the cross-data profiling plot shows the heatmap of the continuous mutation distributions in each dataset (the *x*-axis is the sorted scores, and the tumor types are labeled on the right side).

As the third step, users can select a tumor type and a dataset of interest. Users can explore the dataset by viewing the dataset metadata table or selecting a metadata column and then viewing the corresponding UMAP plot (Figure 2D). The metadata that can be plotted mainly includes cell type annotation, donors, groups assigned by the biological experiment and source (tissue, cell lines, PDX or CTCs). Also, after selecting a mutation type and selecting a specific mutation, users can view the per-data profiling UMAP plot (Figure 2E).

### Explore the mutation-phenotype associations in the single-cell level

**Figure 3.**
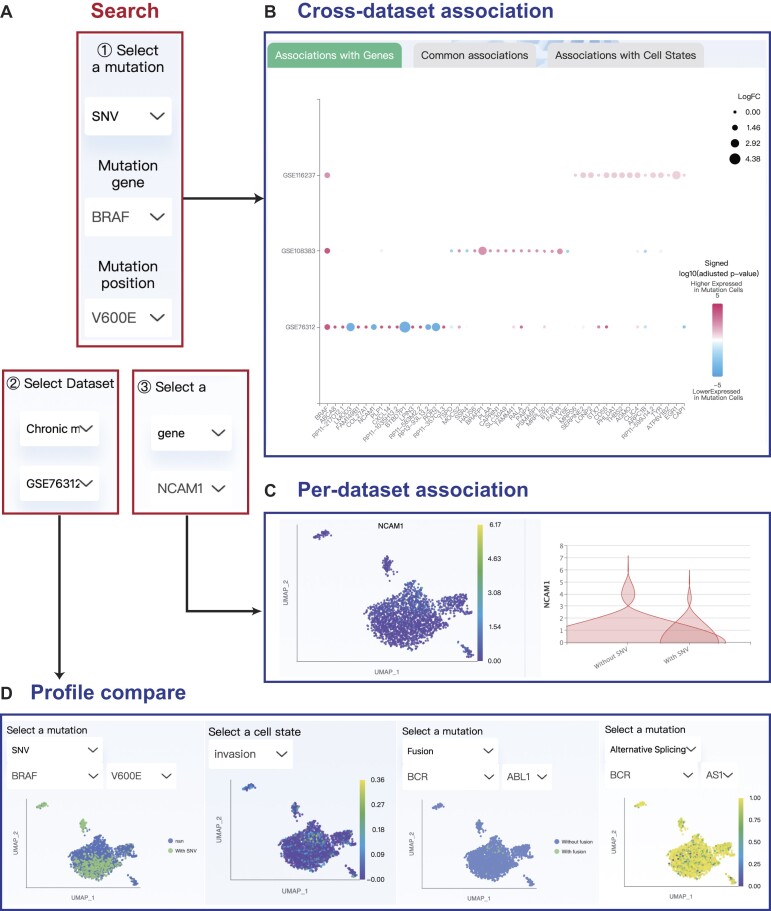
The web interface for cross-dataset and per-dataset mutation-phenotype association in scTML. (**A**) Query interface for selecting a mutation, a dataset and a gene (or a cell state) associated with the mutation. (**B**) The cross-dataset association bubble plot shows the differential analysis results on gene expressions, after selecting the *BRAF* V600E SNV. (**C**) The per-dataset association of *BRAF* V600E SNV and *NCAM1* gene in the GSE76312 dataset, which includes the *NCAM1* UMAP plot and the violin plot comparing *NCAM1* expression levels of cells with and without *BRAF* V600E SNV. (**D**) Multiple mutation and phenotype profiles in the ‘Compare’ page, after selecting the GSE76312 dataset.

On the ‘Association’ page, users can explore the mutation-phenotype associations at the single-cell level. After selecting the mutation of interest (Figure 3A), the cross-dataset association bubble plot shows the differential analysis results on gene expressions or cell state profiles, regarding cells with the selected mutation as one cluster, and cells without the mutation as the other (Figure 3B). The bubble plot shows the top 15 genes with the highest differential analysis significance for each dataset. In the bubble plot, color means direction and dot size means the degree of difference. Also, we require that in the dataset included in the bubble plot, there are at least 10 cells with mutations and at least 10 cells without mutations. For continuous mutations (alternative splicing and CNVs), the plot shows PCCs with gene expressions or cell states.

Users can manually select a dataset of interest (Figure 3A), or click the dataset name on the *x*-axis of the bubble plot, and this dataset will be automatically selected. Then users can explore the association in this dataset. Also, clicking the gene name or cell state name on the y-axis can lead to the gene being automatically selected (Figure 3A and B). After selecting a specific dataset and gene (or cell state), users can further observe the association between the mutation selected earlier and the gene in the dataset, through the gene UMAP plot, the violin plot (or scatter plot for continuous mutations) (Figure 3C).

### Compare and download the landscapes of multiple mutation profiles

The ‘Compare’ page provides a dataset-centric comprehensive presentation and comparison of multiple mutation and phenotype profiles in the selected dataset, considering the first two pages (the ‘Mutation profile’ and ‘Association’ page) both selected a mutation first. Users first select the data type from Smart-seq2 (full-length sequencing), 10X and spatial transcriptomics, then select a specific dataset and view the biological information. Then users can select a specific gene or cell state, and select specific mutations in SNV, fusion, alternative splicing or CNV profiles for viewing and comparing the UMAP plot (Figure 2D). For spatial transcriptomics, scTML provides the tissue images and displays mutation or phenotype information at the corresponding positions of the tissue. This page can be used to compare the biological information, genotype and phenotype UMAP plot, and find the cell subcluster of interest.

On the ‘Download’ page, users can use the data selector to select the tumor type, dataset and specific profile to download. When finding the mutation of interest in the ‘Mutation profile’, users can also jump to the ‘Download’ page by clicking a button and download the corresponding data for custom analysis.

### Usage example

The *BRAF* V600E SNV is an important driver mutation and therapeutic target. Taking *BRAF* V600E as an example, we found that in chronic myeloid leukemia, 22% of cells have *BRAF* V600E mutation (Figure 2C), which is consistent with previous research (50). In the cross-dataset association, we found a negative association between *BRAF* V600E and the *NCAM1* gene. *NCAM1* tends to have a higher expression level in cells without *BRAF* V600E. In the GSE76312 dataset, we further observed that *NCAM1* is mainly expressed in the upper part of the UMAP plot, while the *BRAF* V600E is mainly detected in the UMAP lower part (Figure 3C and D). *NCAM1* (CD56) is found with a link to tumor progression (51,52), can promote leukemogenesis and confer drug resistance in leukemia (53). In the ‘Compare’ page, we also found that mutually exclusive with *BRAF* V600E, *BCR-ABL1* fusion events are mainly detected in the UMAP upper part, and those cells have stronger invasiveness (Figure 3D). Considering the RAS/MAPK pathway (54,55) and *BCR-ABL1* fusion (56) both play important roles in chronic myeloid leukemia, and BRAF is a key factor of the RAS/MAPK pathway (57), our findings indicate the diverse patterns of clonal selection and mutual exclusion of driver mutations in chronic myeloid leukemia.

## Discussion and future directions

We have developed a database scTML by systematically curating published datasets and establishing a comprehensive analyzing framework. scTML includes SNVs, insertions/deletions, gene fusions, alternative splicing and CNVs, along with gene expression, cell states, and other phenotype information from 74 datasets with 2 582 633 cells in total. Providing single-cell datasets with cell-matched multiple mutation types, scTML can also enable researchers online exploring these valuable resources from multiple aspects including cross-dataset and per-dataset profiling, and cross-dataset and per-dataset association.

The three data types of scTML include full-length single-cell transcriptomes with deep sequencing and rich mutation information, as well as 10X single-cell transcriptomics with a large number of cells and cell types, and spatial transcriptomes that provide spatial and tissue slice information. In full-length datasets, we detected 1578.2 mutations per cell on average, while in 10X and spatial transcriptomic datasets, 0.6 and 0.8 mutations were detected per cell, respectively (Supplementary Figure S1A). As for CNVs, due to the full-length transcriptomics covering more genes, greater variations of CNV scores were also observed among full-length datasets (Supplementary Figure S1B). The average variation of CNV scores is 17.8 in full-length datasets, and the average variations are 0.1 and 1.1 in 10X and spatial transcriptomic datasets, respectively.

Wide researchers can be benefited from scTML. Researchers who focus on certain mutations can apply scTML to investigate the distribution of these mutations in different tumors and cell types, study genes and cell states associated with these mutations, and explore whether these mutations are enriched in a specific subcluster of cells (58,59). Oncology researchers can also use scTML to systematically explore how various mutations and molecular mechanisms work together during tumor occurrence and progression, and form inter-tumor and intra-tumor heterogeneity (60,61). The processed data of scTML can also be downloaded and used for the development of new algorithms or computational frameworks. Some high-level analyzing approaches based on mutations can be extended to the single-cell level and bring new discoveries (62–66).

In the future, we will continue to improve and expand scTML. In the current version of scTML, we have collected all full-length datasets with publicly available raw sequencing files, but we have also found that there are some high-quality datasets without publicly available raw sequencing data. In the future, we will strive to contact relevant authors to obtain the raw sequencing data of these datasets. In addition, there are currently more single-cell sequencing technologies with the ability to identify mutations, such as G&T-seq (67) and DR-seq (68). Although the quality and analysis methods of these data are not consistent, including them can further expand our database. In summary, scTML is a valuable resource that provides pan-cancer single-cell mutation landscapes, aiding in the deciphering of heterogeneous cell populations and how mutations shape cell phenotypes.

## Supplementary Material

gkae898_Supplemental_File

## Data Availability

All data and results can be downloaded from the scTML website (http://sctml.xglab.tech/).
