# Supplementary material for: scTML: a pan-cancer single-cell landscape of multiple mutation types
Source: Nucleic Acids Res. 2024 Oct 18;53(D1):D1547–56. doi: 10.1093/nar/gkae898 (PMC11701564; doi:10.1093/nar/gkae898)
Supplement: gkae898_Supplemental_File [file gkae898_supplemental_file.pdf]

scTML: a pan-cancer single-cell landscape of multiple mutation types

Supplementary Materials

Supplementary Tables 1-2

Supplementary Figure 1

Supplementary Table 1. Datasets included in this database

Single-cell sequencing type	Dataset ID	Tumor type	Tumor abbreviation
Smartseq2	GSE118389	Breast Cancer	BRCA
Smartseq2	GSE75688	Breast Cancer	BRCA
Smartseq2	GSE109761	Breast Cancer	BRCA
Smartseq2	GSE153509	Breast Cancer	BRCA
Smartseq2	GSE147356	Breast Cancer	BRCA
Smartseq2	GSE77308	Breast Cancer	BRCA
Smartseq2	GSE123837	Breast Cancer	BRCA
Smartseq2	GSE116237	Melanoma	SKCM
Smartseq2	GSE108383	Melanoma	SKCM
Smartseq2	GSE148345	Colo-Rectal Cancer	CRC
Smartseq2	GSE122582	Colo-Rectal Cancer	CRC
Smartseq2	GSE97693	Colo-Rectal Cancer	CRC
Smartseq2	GSE84465	Glioma	GBM
Smartseq2	GSE57872	Glioma	GBM
Smartseq2	GSE164624	Glioma	LGG
Smartseq2	GSE134269	Glioma	LGG
Smartseq2	GSE154906	Liver cancer	LIHC
Smartseq2	GSE103866	Liver cancer	LIHC
Smartseq2	GSE111894	Lung Cancer	LUAD
Smartseq2	GSE69405	Lung Cancer	LUAD
Smartseq2	GSE117872	Oral squamous cell carcinoma	OSCC
Smartseq2	GSE149041	Oral squamous cell carcinoma	OSCC
Smartseq2	GSE106218	Multiple Myeloma	MM
Smartseq2	GSE118900	Multiple Myeloma	MM
Smartseq2	GSE110499	Multiple Myeloma	MM

Smartseq2	GSE161896	Acute Lymphoblastic Leukemia	ALL
Smartseq2	GSE83142	Acute Lymphoblastic Leukemia	ALL
Smartseq2	GSE81730	Chronic Myelogenous Leukemia	LCML
Smartseq2	GSE76312	Chronic Myelogenous Leukemia	LCML
Smartseq2	GSE81812	Esophageal Cancer	ESCA
Smartseq2	SRP119465	Esophageal Cancer	ESCA
Smartseq2	GSE99795	Prostate cancer	PRAD
Smartseq2	GSE140440	Prostate cancer	PRAD
Smartseq2	GSE113616	Pancreatic Cancer	PAAD
Smartseq2	GSE73121	Renal cell carcinoma	KIRC
10x	GSE135337	Bladder Cancer	BLCA
10x	GSE138709	Biliary Cancer	CHOL
10x	GSE188711	Colo-Rectal Cancer	CRC
10x	GSE182109	Glioma	GBM
10x	GSE163558	Gastric Cancer	STAD
10x	GSE112271	Liver cancer	LIHC
10x	GSE195832	Head and Neck Cancer	HNSC
10x	GSE150825	Head and Neck Cancer	HNSC
10x	GSE172577	Oral squamous cell carcinoma	OSCC
10x	GSE123902	Lung Cancer	LUAD
10x	GSE146100	Lung Cancer	LUAD
10x	GSE176029	Uveal Melanoma	UVM
10x	GSE184880	Ovarian Cancer	OV
10x	GSE154600	Ovarian Cancer	OV
10x	GSE141017	Pancreatic Cancer	PAAD

10x	GSE156405	Pancreatic Cancer	PAAD
10x	GSE157703	Prostate Cancer	PRAD
10x	GSE137829	Prostate Cancer	PRAD
10x	GSE152938	Renal Cell Carcinoma	KIRC
10x	GSE156632	Renal Cell Carcinoma	KIRC
10x	GSE171306	Renal Cell Carcinoma	KIRC
10x	GSE152048	Osteosarcoma	OS
10x	GSE191288	Thyroid Cancer	THCA
Spatial	GSE181300	Head and Neck Cancer	HNSC
Spatial	GSE211956	Ovarian Cancer	OV
Spatial	GSE144239	Skin Squamous Cell Carcinoma	SSCC
Spatial	brca_ffpe_10x_website	Breast Cancer	BRCA
Spatial	cervical_cancer_ffpe_10x_website	Cervical Cancer	CESC
Spatial	crc_ffpe_11mm_10x_website	Colo-Rectal Cancer	CRC
Spatial	crc_ffpe_Xenium_10x_website	Colo-Rectal Cancer	CRC
Spatial	gbm_ffpe_11mm_10x_website	Glioma	GBM
Spatial	intestine_cancer_ffpe_10x_website	Colo-Rectal Cancer	CRC
Spatial	lu_nec_ffpe_11mm_10x_website	Lung Cancer	LNEC
Spatial	lu_scc_ffpe_10x_website	Lung Cancer	LUSC
Spatial	mel_ffpe_10x_website	Melanoma	SKCM
Spatial	ov_ffpe_10x_website	Ovarian Cancer	OV
Spatial	ov_ffpe_11mm_10x_website	Ovarian Cancer	OV
Spatial	pr_acc_ffpe_10x_website	Prostate Cancer	PRAD
Spatial	pr_ffpe_10x_website	Prostate Cancer	PRAD

Supplementary Table 2. Marker genes for cell-type annotation

Cell type	Marker genes
Epithelial cell	*CDH1, MYLK, ANKRD30A, ABCB10, SFTPC, EPCAM, FOLH1, KLK3, KRT8, KRT18, KRT19*
Fibroblast	*COL1A1, COL3A1, THY1, NECTIN1, FAP, PTPN13, C5AR2, LRP1*
B/Plasma cell	*CD19, MS4A1, BANK1, BLK, AIM2, MZB1, CD38, AC026202.3, JSRP1, LINC00582, PARM1, TAS1R3*
T/NK cell	*CD3D, CD3G, CD3E, NKG7, GZMB*
Macro/Mono/DC	*CD68, CD14, MRC1, CD93, CREM, CSF1R, CCL18, ICAM4, ACPP, ADGRE2, ADGRE3, CD209, CD83, CD1A*
Endothelial cell	*VWF, PECAM1, CDH5, VEGFA, FLT1, ECSCR, ADGRL2, SELE, ICAM1*
Neuron	*STMN2, RBFOX3, CSF3, DLG4*
Oligodendrocyte	*MOG, OLIG1, OLIG2, PDGFRA, PLP1, MBP, MAG, SOX10*
Astrocyte	*AGXT2L1, GFAP, ALDOC, AGT, ALDH1L1*
Melanocyte	*MLANA, PMEL, MITF, TYR, S100A4, CDC42EP3, S100B, DCT, IGFBP7*
Hepatocyte	*ALB, FGG, FGA, HPX*
Hematopoietic stem cell (HSC)	*CD34, ITGA5, PROM1, CD164, THY1, ALDH1A1*

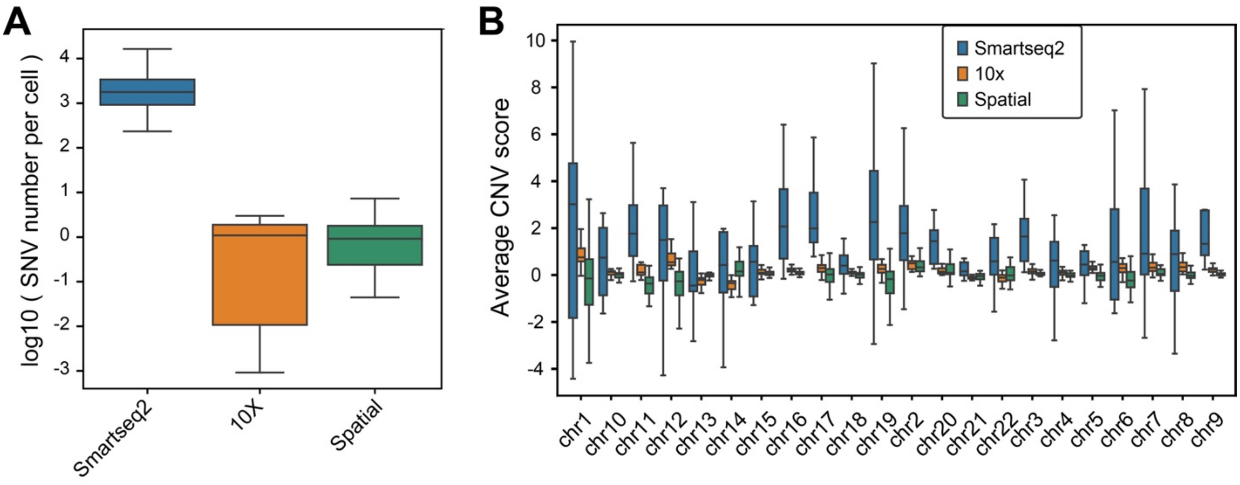

Supplementary Figure 1. Comparing mutations in different single-cell and spatial platforms.

(A) The logarithmical average number of mutations per cell in each dataset from Smartseq2, 10X, and Spatial transcriptomic platforms. **(B)** The average chromosome-level CNV scores in each dataset from Smartseq2, 10X, and Spatial transcriptomic platforms. We calculated the average CNV score of all segments on a chromosome, and then obtained the average CNV score of all cells in each dataset at the chromosome level. Greater variations of CNV scores were also observed among Smartseq2 datasets.
